# Multi-stimuli responsive Cu-MOFs@Keratin drug delivery system for chemodynamic therapy

**DOI:** 10.3389/fbioe.2023.1125348

**Published:** 2023-02-02

**Authors:** Jinsong Du, Guanping Chen, Xinyi Yuan, Jiang Yuan, Li Li

**Affiliations:** ^1^ Department of Biochemistry and Molecular Biology, School of Basic Medical Sciences and the Affiliated Hospital, Key Laboratory of Aging and Cancer Biology of Zhejiang Province, Hangzhou Normal University, Hangzhou, China; ^2^ Jiangsu Key Laboratory of Bio-functional Materials, School of Chemistry and Materials Science, Nanjing Normal University, Nanjing, China; ^3^ Cancer Institute of Integrated Traditional Chinese and Western Medicine, Zhejiang Academy of Traditional Chinese Medicine, Tongde Hospital of Zhejiang Province, Hangzhou, China; ^4^ School of Clinical Medicine and The Affiliated Hospital, Hangzhou Normal University, Hangzhou, China

**Keywords:** keratin, MOFs, stimuli-responsive, chemodynamic therapy, drug delivery system

## Abstract

Although the potential of metal-organic framework (MOF) nanoparticles as drug delivery systems (DDS) for cancer treatment has been established by numerous studies, their clinical applications are still limited due to relatively poor biocompatibility. We fabricated a multifunctional Cu-MOFs@Keratin DDS for loaded drug and chemodynamic therapy (CDT) against tumor cells. The Cu-MOFs core was prepared using a hydrothermal method, and then loaded with the anticancer drug DOX and wrapped in human hair keratin. The Cu-MOFs@Keratin was well characterized by transmission electron microscopy (TEM), fourier transform infrared spectroscopy (FTIR), dynamic light scattering (DLS), and X-ray photoelectron spectroscopy (XPS). Characterization and pharmacokinetic studies of Cu-MOFs@Keratin were performed *in vitro* and *in vivo*. The keratin shell reduced the cytotoxicity and potential leakage of Cu-MOFs to normal cells, and allowed the drug-loaded nanoparticles to accumulate in the tumor tissues through enhanced permeability and retention effect (EPR). The particles entered the tumor cells *via* endocytosis and disintegrated under the stimulation of intracellular environment, thereby releasing DOX in a controlled manner. In addition, the Cu-MOFs produced hydroxyl radicals (·OH) by consuming presence of high intracellular levels of glutathione (GSH) and H_2_O_2_, which decreased the viability of the tumor cells.

## 1 Introduction

Crystalline porous metal-organic frameworks (MOFs) with periodic network structure are formed by self-assembly of transition metal ions and organic ligands. MOFs can be easily functionalized, due to their adjustable particle size, high porosity and high specific surface area (Seo et al., 2000; Oh and Mirkin, 2005; Chen et al., 2009; Yan et al., 2022). Since Horcajada et al. tested MOFs as drug delivery systems (DDS) for the first time in 2008, there have been consistent efforts to improve their efficacy by devising strategies to avoid possible drug leakage (Horcajada et al., 2008; Pandey et al., 2020). Several MOFs have been developed in recent years as DDS for tumor therapy (Zheng et al., 2021). Nevertheless, the poor biocompatibility of MOFs is a major obstacle to their clinical translation (Chen et al., 2021).

MOFs can be coated with biomaterials to improve their biocompatibility and stability in the bloodstream, and prevent premature drug release (Wuttke et al., 2015). Human hair keratin is an ideal shell for MOFs due to its biodegradability and stability under normal physiological conditions. Compared with PEG and zwitterionic polymer, human hair keratin can effectively avoid the immune response and allergic reactions (Du et al., 2021a). On the other hand, it can easily disintegrate in the tumor microenvironment (TME) in response to low pH, and high levels of glutathione (GSH) and trypsin, which is conducive to release of the drug cargo in the tumor tissues (Du et al., 2020). Wang et al. fabricated a DDS consisting of sulfhydryl-linked keratin, which was highly stable in a simulated blood environment (Wang et al., 2020). Furthermore, Han et al. coated drug-loaded nanoparticles with keratin for targeted release in the TME (Han et al., 2020). In a previous study, we used keratin to package drug-loaded mesoporous silica nanoparticles, and found that the keratin shell prevented drug leakage in a simulated physiological environment (Du et al., 2021b). Therefore, coating MOFs with keratin could enhance their stability in the bloodstream, strengthen their stimuli responsiveness and biocompatibility.

Chemodynamic therapy (CDT) is based on the conversion of high level hydrogen peroxide (H_2_O_2_) into hydroxyl radicals (·OH) by Fenton/Fenton-like reactions in the TME (Feng et al., 2019; Huo et al., 2019). In brief, multivalent ion (Fe^2+/3+^ and Cu^1+/2+^) can simulate enzymatic catalysis of H_2_O_2_ into OH radicals, a highly toxic reactive oxygen species (ROS) that eventually kills the tumor cells (Jiang et al., 2019). While the Cu-catalyzed Fenton-like reaction is highly efficient in neutral and weakly acidic media, and is kinetically and energetically advantageous than that with Fe^2+^(Garcia-Bosch and Siegler, 2016; Wang et al., 2019; Wang and Song, 2020). Using polydopamine as a stabilizer and binder, An et al. designed a nano-catalytic system with Cu^2+^-doped ZIF-8 for CDT, which reduced the intracellular levels of GSH and caused the generation of cytotoxic ROS *via* a Cu^+^-mediated Fenton-like reaction (An et al., 2020). Likewise, Liu et al. prepared biodegradable copper-manganese silicate nanospheres for chemodynamic and photodynamic therapy of tumors, which represented good biocompatibility and achieved exceptional cancer therapeutic effects *in vitro* and *in vivo* (Liu et al., 2019). Thus, Cu^2+^-directed CDT provides a new potential approach for tumor treatment.

**FIGURE 1:**
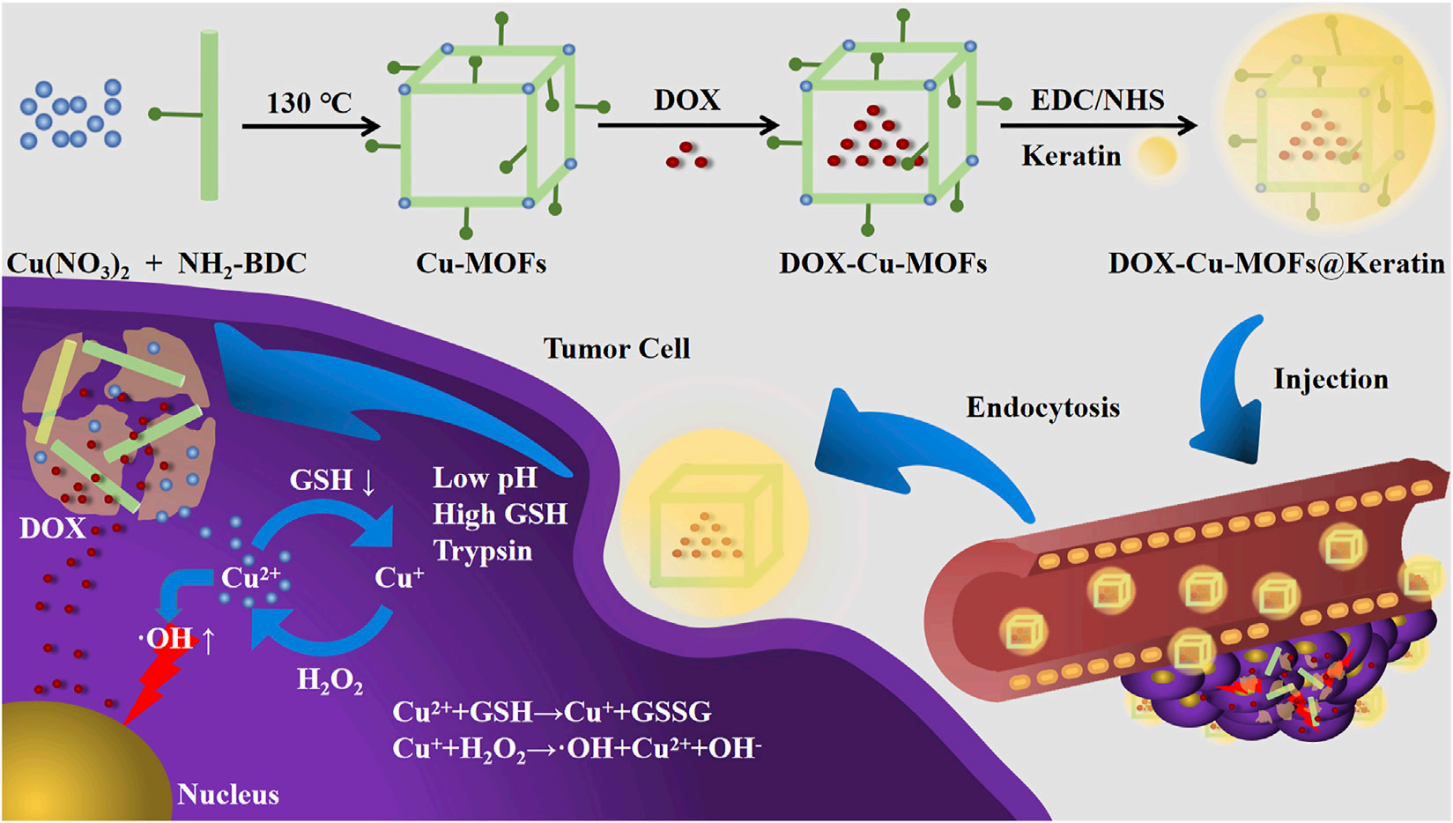
Schematic diagram of the preparation and working flowchart of DOX-Cu-MOFs@Keratin.

Doxorubicin (DOX) is a commonly used chemotherapy drug in cancer treatment (Zheng et al., 2016; Chen et al., 2017). However, drug monotherapies are largely ineffective since the unique characteristics of TME allow the tumor cells to rapidly proliferate and metastasize, and develop resistance against the chemotherapy agents (Chang et al., 2019). Notably, Cu^2+^-directed CDT can also augment the effects of chemotherapy drugs by damaging the TME. In this study, we prepared stimuli-responsive, biocompatible keratin-coated Cu-MOFs (Cu-MOFs@Keratin) with capacity for drug loading and CDT, and tested its performance as a DDS for the anticancer drug DOX (Figure 1). Cu-MOFs bearing amino groups were synthesized by the hydrothermal method, loaded with DOX and coated with keratin through lactamization. The diameter of the particles was conducive to their accumulation in the tumor tissues through enhanced permeability and retention effect (EPR) (Gerlowski and Jain, 1986; Maeda et al., 2000). Following endocytosis by the tumor cells, the keratin coating was destroyed in response to the low pH, and high levels of GSH and trypsin. The Cu-MOFs also disintegrated in the presence of GSH and released DOX, and generated OH radicals through Fenton-like reaction as already described. The Cu-MOFs@Keratin effectively inhibited tumor cell growth *in vitro* and *in vivo*, it could be used for synergistic chemotherapy and CDT against cancer. Overall, we prepared an efficient and multi-stimuli responsive drug delivery system using keratin-coated Cu-MOFs, which endows improved biocompatibility, reduced systemic toxicity and good anti-tumor efficacy for DOX. The results of this study provides a new approach for constructing effective Cu-MOFs@Keratin nanoplatform for synergistic chemotherapy and chemodynamic therapy against tumor cells.

## 2 Materials and methods

### 2.1 Materials

Doxorubicin hydrochloride (DOX) was purchased from Hisun Pharmaceutical Co., Ltd. (Zhejiang, China). 5,5′-Dithiobis-(2-nitrobenzoic acid) (DTNB) and Glutathione (GSH) were provided by Aladdin Reagent Co., Ltd. (Shanghai, China). Other chemicals were analytical grade and were used directly.

### 2.2 Preparation of Cu-MOFs@Keratin

Cu-MOFs containing amino groups were prepared by a hydrothermal method as previously described (Zhang et al., 2019). Briefly, 1.2g PVP was dissolved in a mixed solution of 24 mL ethanol and 24 mL DMF, and 36 mg 2-aminoterephthalic acid (NH_2_-BDC) and 144 mg cupric nitrate were dissolved in 48 mL ethanol. Both solutions were mixed and transferred into a high-pressure reactor, and heated at 130°C for 6 h. The product was collected by centrifugation, and washed with ethanol and deionized water (DI). The keratin containing of numerous carboxyl groups was prepared according to the method previously reported by our group (Du et al., 2020). Then, 40 mg Cu-MOFs containing amino groups, 40 mg keratin containing carboxyl groups, 10 mg EDCHCl and 6 mg NHS were mixed in 16 mL DI and stirred at constant speed for 4 h. The reaction was then terminated and the solution was centrifuged at 10,000 rpm for 10 min, and the pellet was freeze-dried.

### 2.3 Preparation of DOX-loaded Cu-MOFs@Keratin

To load DOX, 20 mg of the drug was mixed with 10 mg Cu-MOFs in 10 mL DI. After adding 40 μL triethylamine, the mixture was allowed to react for 48 h and then centrifuged at 10000 rpm for 10 min. The pellet was washed several times with DI as above and freeze-dried. The keratin was coated as described in the previous section.

### 2.4 GSH depletion assay

Cu-MOFs of different weights were incubated in GSH solution for 3 h and centrifuged. The clear supernatant was then mixed with the same volume of DTNB and the absorbance at 325 nm and 412 nm was measured after 5 min. GSH consumption by Cu-MOFs@Keratin was similarly tested. In addition, the same weight of Cu-MOFs@Keratin was incubated with GSH solutions at different pH for 3 h, and the absorbance was measured as described above.

### 2.5 ROS (·OH) generation

GSH solutions containing the same amount of Cu-MOFs or Cu-MOFs@Keratin were incubated with different concentrations of H_2_O_2_ and 6 μg/mL MB for 3 h. The solutions were centrifugated and the absorbance of the supernatants was measured at 664 nm.

### 2.6 Drug release

DOX-Cu-MOFs@Keratin was incubated in simulated TME consisting of 10 mM GSH and 1 mg/mL trypsin at low pH (5). Drug release curve were measured as previously described (Du et al., 2021a).

### 2.7 *In vitro* cytotoxicity

MTT assay was used to test the cytotoxicity of Cu-MOFs, Cu-MOFs@Keratin and DOX-Cu-MOFs@Keratin in L929 and A549 cells. L929 cells in the logarithmic growth phase were seeded in a 96-well plate at the density of 1×10^5^ cells/mL and incubated for 12 h. Different concentrations of the respective particles were added in 100 μL medium, and each dose was tested in sextuplet. After 48 h of culture, the medium was discarded, and 100 µL MTT reagent and 900 µL medium was added to each well. The cells were incubated for 4 h, and the reaction was terminated with 100 μL DMSO. After 30 min of constant shaking to dissolve the formazan crystals, the absorbance of the wells was measured at 490 nm using a microplate analyzer. The survival rates of the cells were calculated using the relative ratio between absorbance.

### 2.8 Cell uptake

A549 cells in their logarithmic growth stage were incubated with DOX-Cu-MOFs@Keratin for 4, 14 and 24 h, and washed thrice with PBS after discarding the medium. The cells were fixed with 4% glutaraldehyde for 10 min, washed twice with PBS and stained with DAPI for 15 min. Following three washes with PBS, the cells were observed and photographed by confocal laser scanning microscopy (CLSM).

### 2.9 Animal experiment

Male C57BL/6 mice (initial body weight about 18–20 g) were obtained from Beijing Vital River Laboratory Animal Technologies Co. Ltd. and housed under SPF conditions with controlled temperature (18°C–22°C), humidity (30%–60%), and a 12 h light/dark cycle. To establish tumors, 1×10^6^ Hepa1-6 cells mixed with Matrigel (1:1) were subcutaneously injected in the right flank area of mice. Seven days after implantation, the mice were randomized into the control, free DOX and DOX-Cu-MOFs@Keratin groups (*n* = 5 each). The mice in the treatment groups were injected intraperitoneally with 5 mg/kg (0.2 mL) DOX or the equivalent amounts of DOX-Cu-MOFs@Keratin every other day, and control mice received the same volume of normal saline. The mice were weighed every day to evaluate the adverse effects of the drugs. Tumor volume was calculated as (L×W^2^)/2, where L is the diameter of the longest dimension and W is the orthogonal diameter. At the end of the experiment, the animals were euthanized by cervical dislocation, and the tumor tissue was removed and weighed. The remaining tissues were frozen in liquid nitrogen for subsequent analysis.

### 2.10 Statistical analysis

The results are expressed as the mean ± standard deviation (SD). Statistical analysis was performed using Origin 9.0 (OriginLab, Northampton, MA). Significant differences were determined using one-way ANOVA. *p* < 0.05 was considered statistically significant.

## 3 Results and discussion

### 3.1 Preparation and characterization of Cu-MOFs@Keratin

**FIGURE 2:**
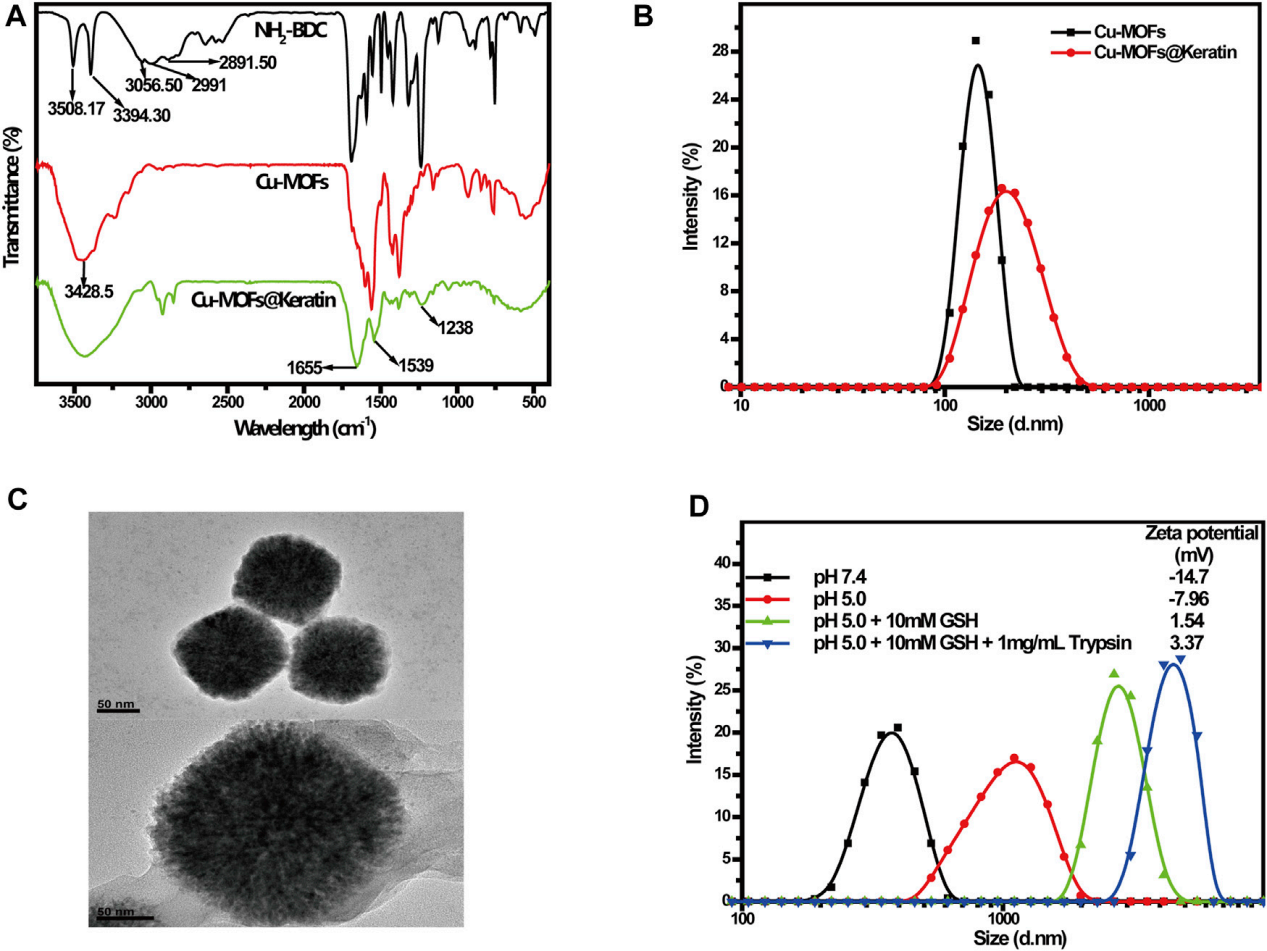
**(A)** FT-IR spectra of NH_2_-BDC, Cu-MOFs and Cu-MOFs@Keratin; **(B)** Particle size distribution of Cu-MOFs and Cu-MOFs@Keratin; **(C)** TEM of Cu-MOFs (top) and Cu-MOFs@Keratin (bottom); **(D)** Particle size distribution and Zeta potential of Cu-MOFs@Keratin in different simulation environments.

Cu-MOFs@Keratin was prepared through amidation reaction, and NH_2_-BDC, Cu-MOFs and Cu-MOFs@Keratin were characterized through FT-IR spectroscopy (Figure 2A). As compared to the spectrum of NH_2_-BDC, the peak at 2991 cm^−1^ representing the carboxyl group was absent in the Cu-MOFs spectra, indicating successful coordination between Cu^2+^ and the carboxyl group of NH_2_-BDC. Notably, the shift of absorption peaks from 3508.17 cm^−1^ and 3394.3 cm^−1^ to 3428.5 cm^−1^ indicated the formation of hydrogen bonds with stable skeleton structure in the MOFs. In the spectra of Cu-MOFs@Keratin, the absorption peaks at 1655 cm^−1^, 1539 cm^−1^ and 1238 cm^−1^ were corresponding to keratin amide I, II and IIIbonds, respectively, indicating that the Cu-MOFs were successfully encapsulated within the keratin shell.

Subsequent DLS analyses showed that the average particle size of Cu-MOFs and Cu-MOFs@Keratin were 167.1 ± 4.3 nm and 201.5 ± 3.2 nm, respectively, which is within the expectation that the size of the particles increased following keratin coating (Figure 2B). The TEM images further showed the uniform size of Cu-MOFs, and a translucent shell of the Cu-MOFs@Keratin that was resulted from keratin coating (Figure 2C). Furthermore, incubating Cu-MOFs@Keratin in 10% FBS for 10 days did not alter the particle size (Supplementary Figure S1), demonstrating its good stability in a simulated physiological environment. The favorable particle size and serum stability of Cu-MOFs@Keratin is beneficial for their long-term retention in the bloodstream and subsequent EPR.

The TME is characterized by low pH and high levels of GSH and trypsin. To assess whether the keratin coating enhanced the responsiveness of the encapsulated Cu-MOFs to the above stimuli, we exposed the particles to different pH, GSH and trypsin levels that simulated the physiological and tumor-specific conditions. As shown in Figure 2D, lower pH and high levels of GSH increased the particle size and Zeta potentials of Cu-MOFs@Keratin. Further addition of trypsin maximized both the particle size and Zeta potentials. As Keratin is protonated in an acidic environment, its disulfide and peptide bonds are easily broken in the presence of GSH and trypsin, respectively, which further results in disintegration of the coating and increase of the particle size and Zeta potentials (Du et al., 2020). Therefore, the Cu-MOFs@Keratin was effectively responsive to the stimulus of the TME, which was attributed from the biochemical characteristics of the keratin shell in the physiological environment.

**FIGURE 3:**
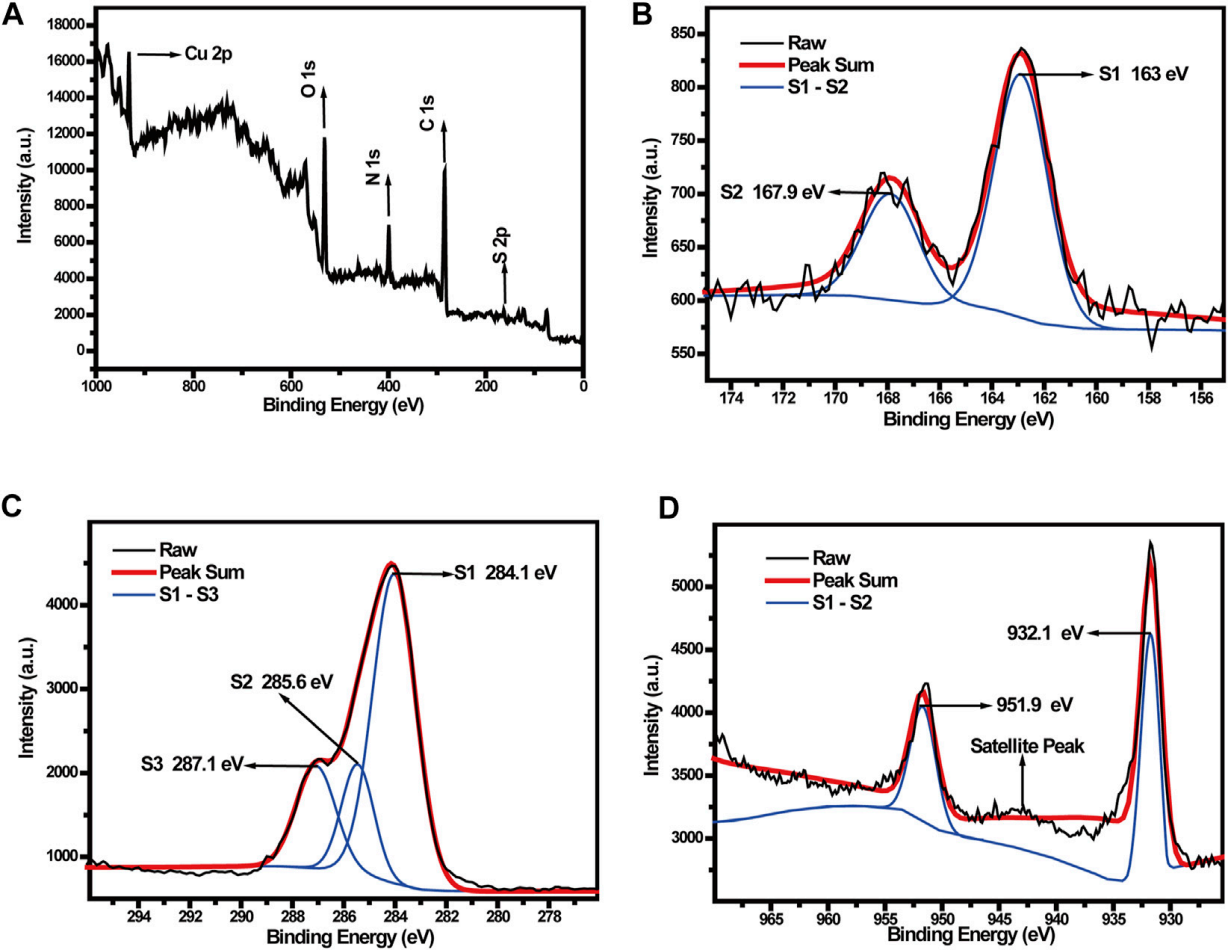
**(A)** XPS survey spectra and **(B)** S, **(C)** C, **(D)** Cu element XPS spectrum of Cu-MOFs@Keratin.

To analyze the composition and valence states of the elements, and functional groups in the Cu-MOFs@Keratin particles, X-ray photoelectron spectroscopy (XPS) was performed. As shown in Figure 3A, the full XPS spectrum of Cu-MOFs@Keratin revealed presence of the elements S, C, N, O and Cu. The peak-differentiation-imitating diagram of S element showed characteristic peaks at 163 eV and 167.9 eV (Figure 3B). The binding energy at 162.8 eV–163.5 eV corresponded to mercaptan substances, indicating the presence of keratin. The XPS peak fitting diagram of element C showed characteristic peaks at 284.1 eV, 285.6 eV and 287.1 eV (Figure 3C). Considering that the electron binding energy of C was about 286.8 eV after combining with carbonyl oxygen, and was above 288 eV after combining with carbonyl oxygen and hydroxyl (Siokou et al., 2011; Zhu et al., 2016; Fu et al., 2020). The XPS peaks of the element C represented the presence of numerous carbonyl groups but absence of carboxyl groups, which was in consistence with the FT-IR spectroscopic analyses that the carboxyl-containing keratin and amino-containing Cu-MOFs were successfully linked by amidation. Especially, the XPS peak fitting diagram of Cu element indicated that the characteristic peaks were at 932.1 and 951.9 eV, which were corresponding to the Cu 2p_3/2_ and Cu 2p_1/2_, respectively. Appearance of the satellite peak between the two characteristic peaks indicated that Cu has a valence state of +2 (Figure 3D), which could react with GSH to catalyze the conversion of H_2_O_2_ into OH radicals (Liu et al., 2019).

### 3.2 GSH depletion and ROS generation

**FIGURE 4:**
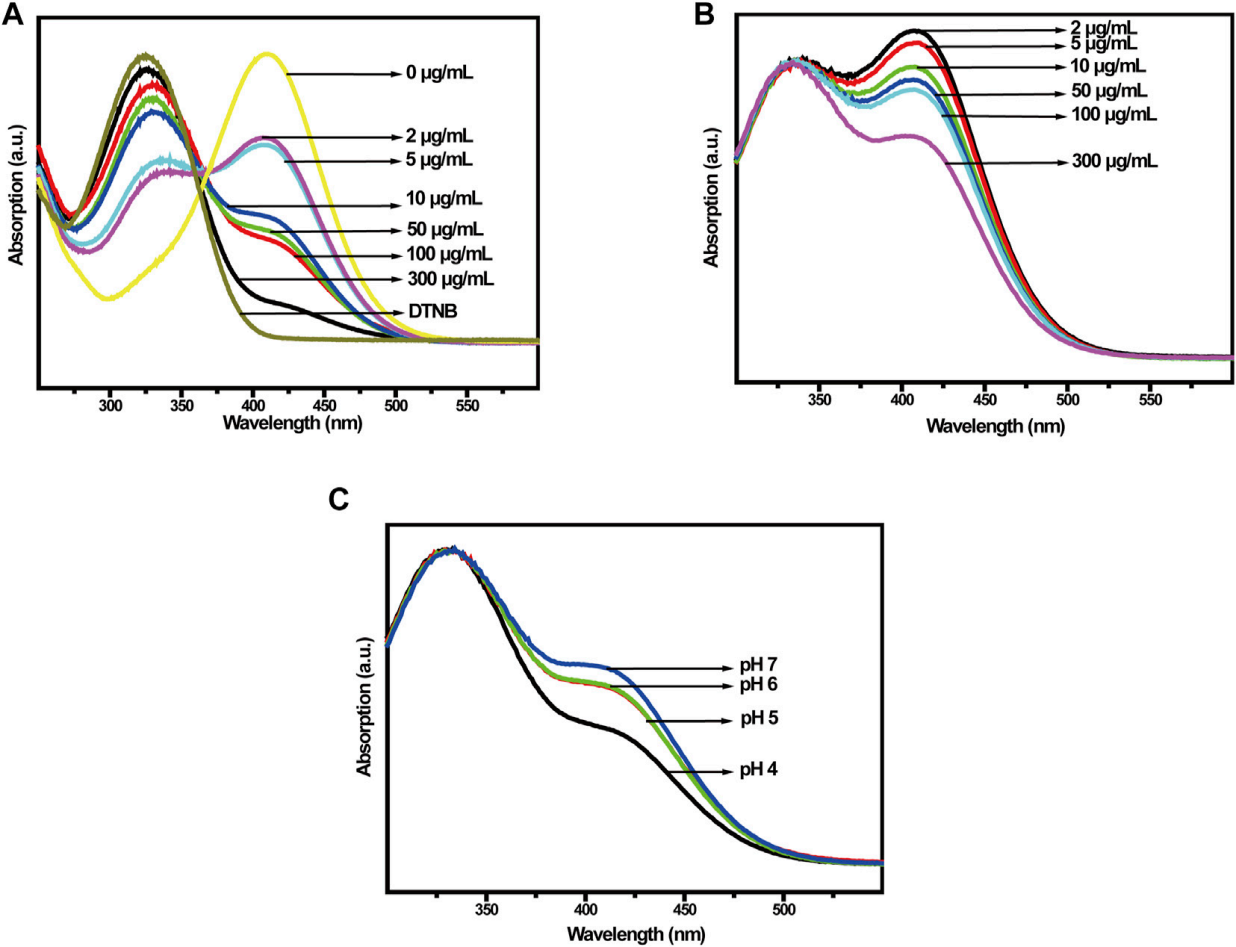
**(A)** UV-Vis spectra of Cu-MOFs and **(B)** Cu-MOFs@Keratin co-incubated with GSH at different concentrations; **(C)** UV-Vis spectra of GSH with different pH values after co-incubation with Cu-MOFs@Keratin.

In the TME, the Cu-MOFs@Keratin could initially react with antioxidant GSH, inducing GSH depletion and Cu^+^ generation (Poyton et al., 2016). Therefore, we measured the ability of Cu-MOFs and Cu-MOFs@Keratin to oxidize GSH using the DTNB probe. As shown in Figure 4A, with the increase in the concentrations of Cu-MOFs, the absorption peak of GSH at 412 nm was decreased, while that of DTNB at 325 nm was increased, indicating that Cu-MOFs can effectively consume GSH. Likewise, Cu-MOFs@Keratin also quenched the levels of GSH in a concentration-dependent manner (Figure 4B). Furthermore, the ability of Cu-MOFs@Keratin in consuming the GSH was enhanced in an acidic environment, which could be attributed to the disintegration of the keratin shell and the increased accessibility of the Cu-MOFs to GSH (Figure 4C). Therefore, the Cu-MOFs@Keratin particles can directly disrupt the TME by depleting GSH, oxidation of GSH generating Cu^+^ that further breaks down H_2_O_2_ through Fenton-like reaction to produce high levels of the OH radical (Garcia-Bosch and Siegler, 2016; Wang et al., 2019).

**FIGURE 5:**
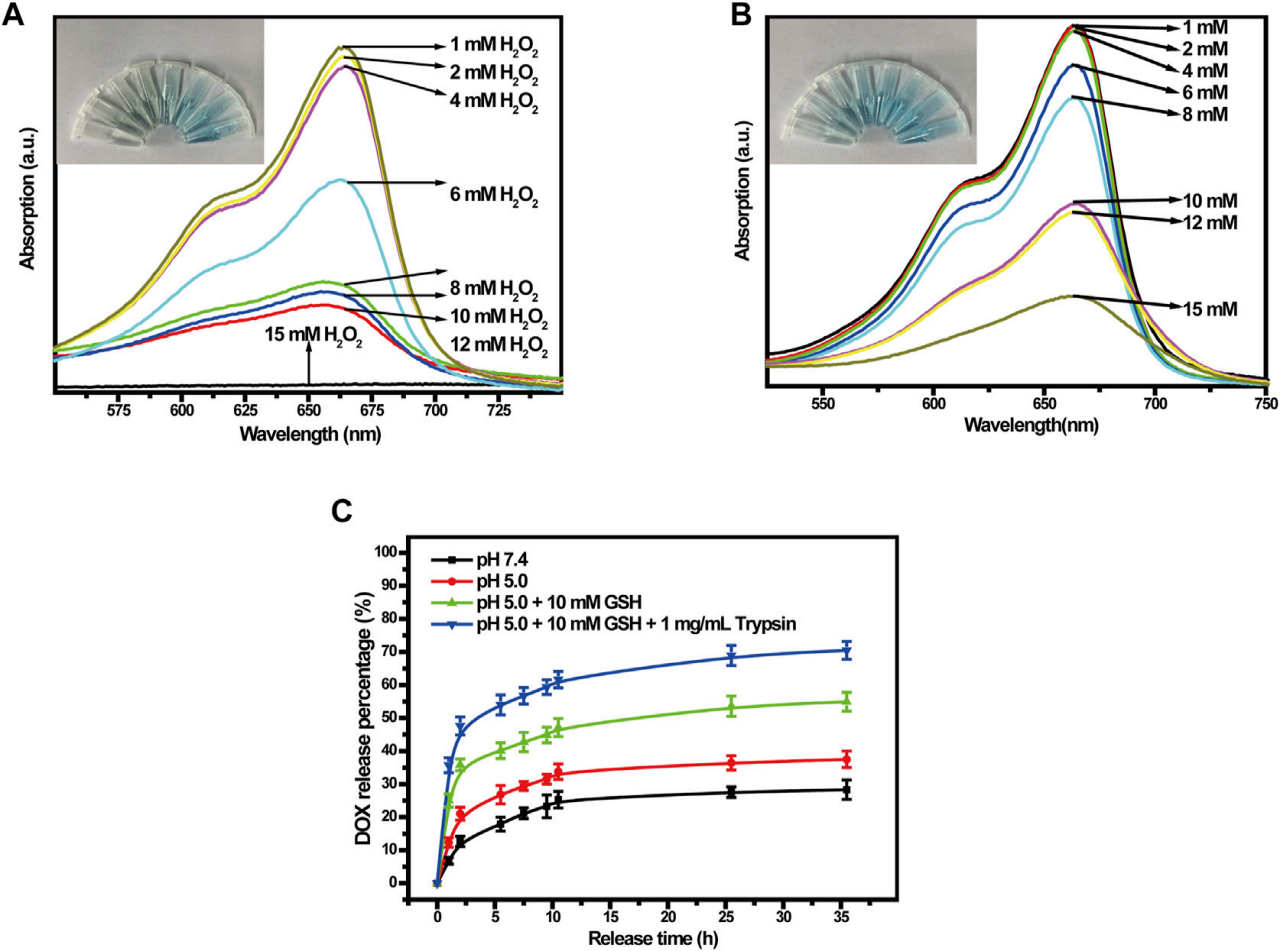
**(A)** UV-Vis spectra of different concentrations of H_2_O_2_ co-incubated with Cu-MOFs and **(B)** Cu-MOFs@Keratin GSH solutions. Cu-MOFs and Cu-MOFs@Keratin can decompose H_2_O_2_ to generate OH through a catalytic Fenton-like reaction. To assess the generation of OH, MB with maximum absorption wavelength at 664 nm was used as an indicator, which could be bleached by OH from dark blue to white; **(C)** Cumulative release curves of DOX-Cu-MOFs@Keratin.

To verify the generation of ROS, we used methylene blue (MB) to track OH production by Cu-MOFs and Cu-MOFs@Keratin in the presence of GSH and H_2_O_2_. The oxidation of MB by OH bleaches the dye from dark blue to white, which could be detected by an absorption decrease of MB at 664 nm. As shown in Figure 5A, addition of H_2_O_2_ decreased the absorption intensity of MB in a concentration-dependent manner in the presence of Cu-MOFs and GSH, which can be attributed from the generation of OH through Cu^+^-breakdown of H_2_O_2_. In addition, the catalytic activity of Cu-MOFs was unaffected by the keratin coating, the absorbance of MB was also decreased in the presence of Cu-MOFs@Keratin and GSH, upon addition of varied concentrations of H_2_O_2_ (Figure 5B), indicating that Cu-MOFs@Keratin can also generate OH in the TME through Fenton-like reaction.

### 3.3 *In vitro* drug release

To test the performance of Cu-MOFs@Keratin, we loaded the chemotherapy drug DOX in the Cu-MOFs (Supplementary Figure S2), with the drug loading rate and encapsulation rate being kept at 27.1% ± 2.6% and 75.3% ± 3.1%, respectively (Supplementary Figure S3). We next analyzed the rate of drug release from DOX-Cu-MOFs@Keratin in different simulated environments using the dialysis method. As shown in Figure 5C, the drug release rate of DOX-Cu-MOFs@Keratin was dependent on the pH and GSH level. In the simulated normal physiological tissue (pH 7.4), DOX release rate of 28.29% indicated that the DDS was relatively stable at physiological pH. Especially, the DOX release rate increased at low pH and high levels of GSH and trypsin, with the highest release rate reached 70.47%. These results indicated that DOX-Cu-MOFs@Keratin can effectively release the drug cargo in the TME and prevent drug leakage in the physiological environment.

### 3.4 *In vitro* cytotoxicity

**FIGURE 6:**
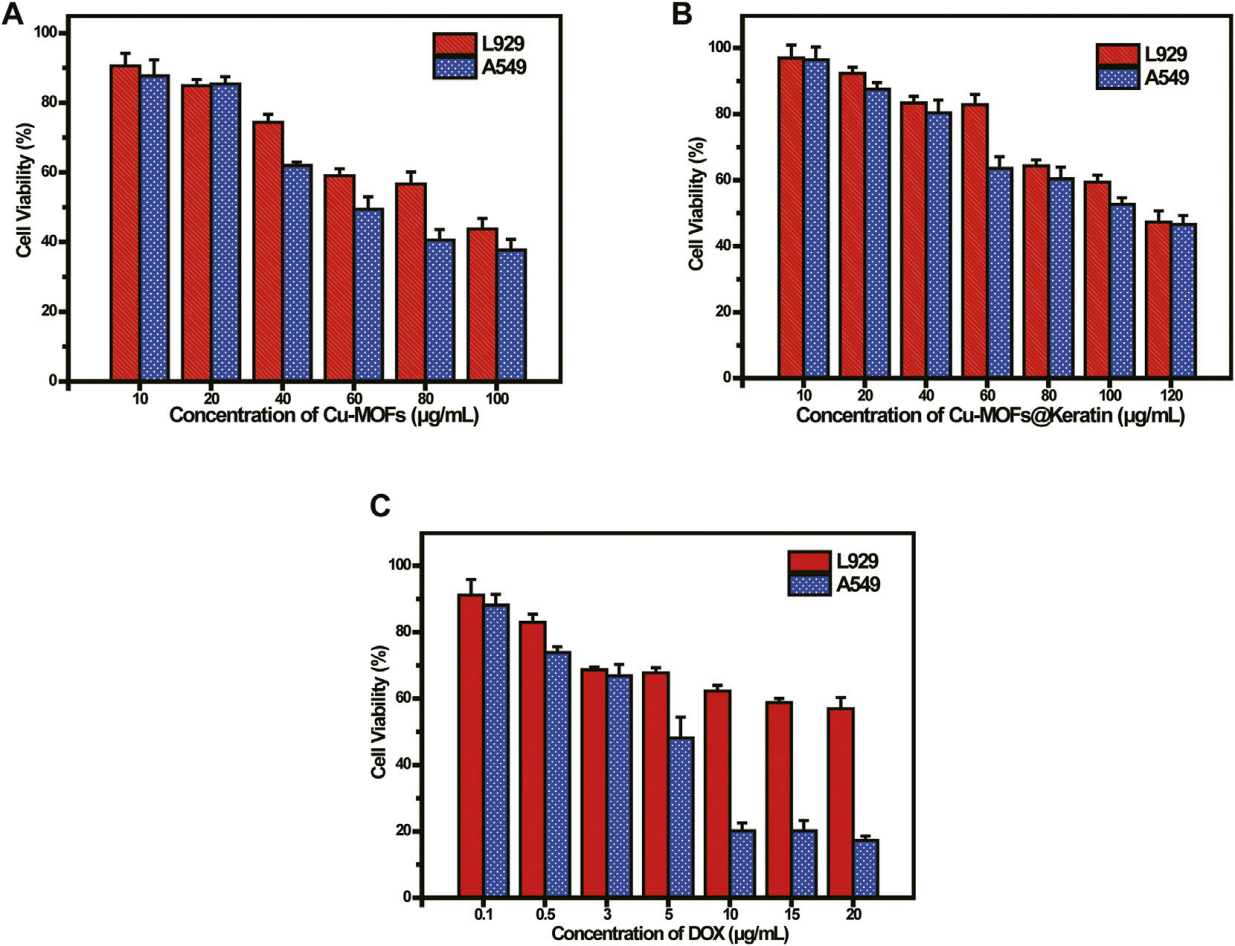
**(A)** Viability of L929 and A549 cells cultured with Cu-MOFs and **(B)** Cu-MOFs@Keratin after 48 h incubation; **(C)** Viability of L929 and A549 cells cultured with DOX-Cu-MOFs@Keratin and DOX after 48 h incubation.

To evaluate the biocompatibility and anti-tumor effects of Cu-MOFs, we tested the viability of mouse embryonic fibroblast (L929) cells and human non-small cell lung cancer (A549) cells incubated with the particles. As shown in Figure 6A, the overall survival rate of the A549 cells was lower than that of the L929 cells. As already mentioned, the Cu-MOFs generated high levels of OH in the presence of GSH and H_2_O_2_, which has a cytotoxic effect. As shown in Figure 6B, more than 80% of the L929 cells survive following exposure to 60 μg/mL Cu-MOFs@Keratin, which was significantly higher than that observed with the Cu-MOFs. Furthermore, encapsulation by Cu-MOFs@Keratin protected the normal cells from the cytotoxic effects of DOX, but effectively inhibited the growth of tumor cells (Figure 6C). These results demonstrated that coating the Cu-MOFs with the keratin shell improved their biocompatibility while retaining the anti-tumor effects of DOX and Cu-MOFs.

### 3.5 Cellular uptake assay

**FIGURE 7:**
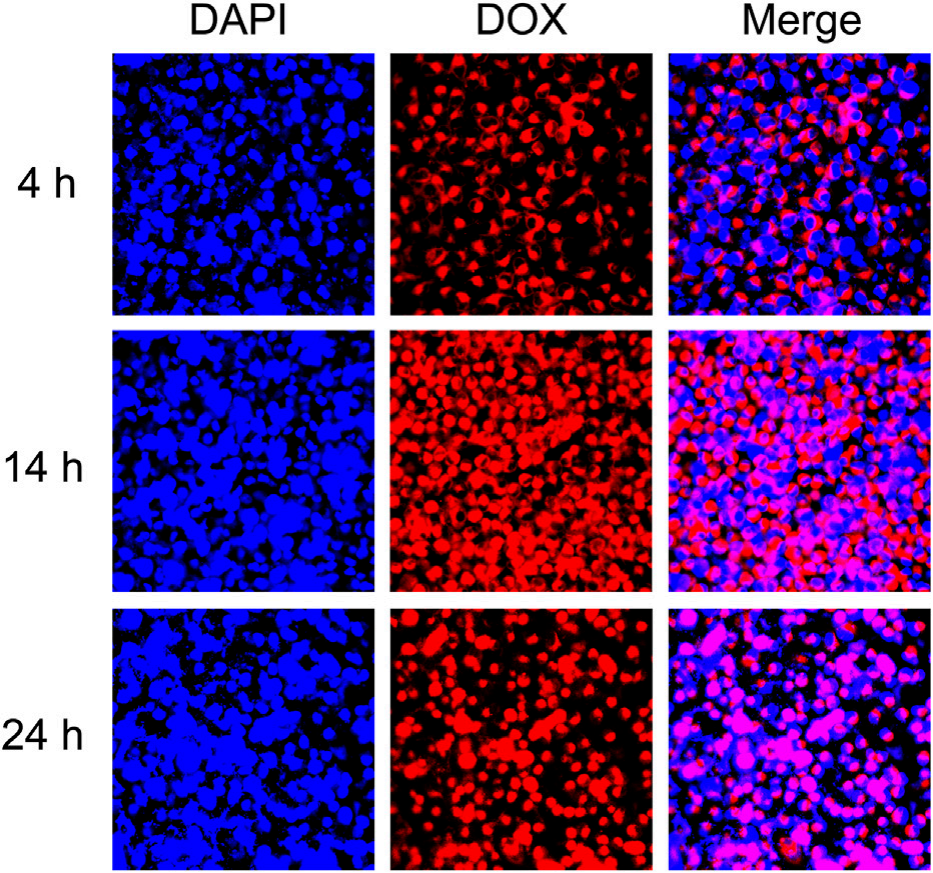
CLSM images of DOX-Cu-MOFs@Keratin.

To verify the uptake of DOX-Cu-MOFs@Keratin, the tumor cells were incubated with DOX-Cu-MOFs@Keratin and observed by CLSM. As shown in Figure 7, the tumor cells emitted the red fluorescence of DOX following 4 h of incubation with DOX-Cu-MOFs@Keratin, which intensified with longer exposure to the nanoparticles, and almost eclipsed the blue fluorescence of DAPI (nucleus) after 24 h. These results indicated that DOX-Cu-MOFs@Keratin can enter tumor cells through endocytosis and release drugs into the nucleus of the tumor cells.

### 3.6 Inhibited tumor growth *in vivo*

**FIGURE 8:**
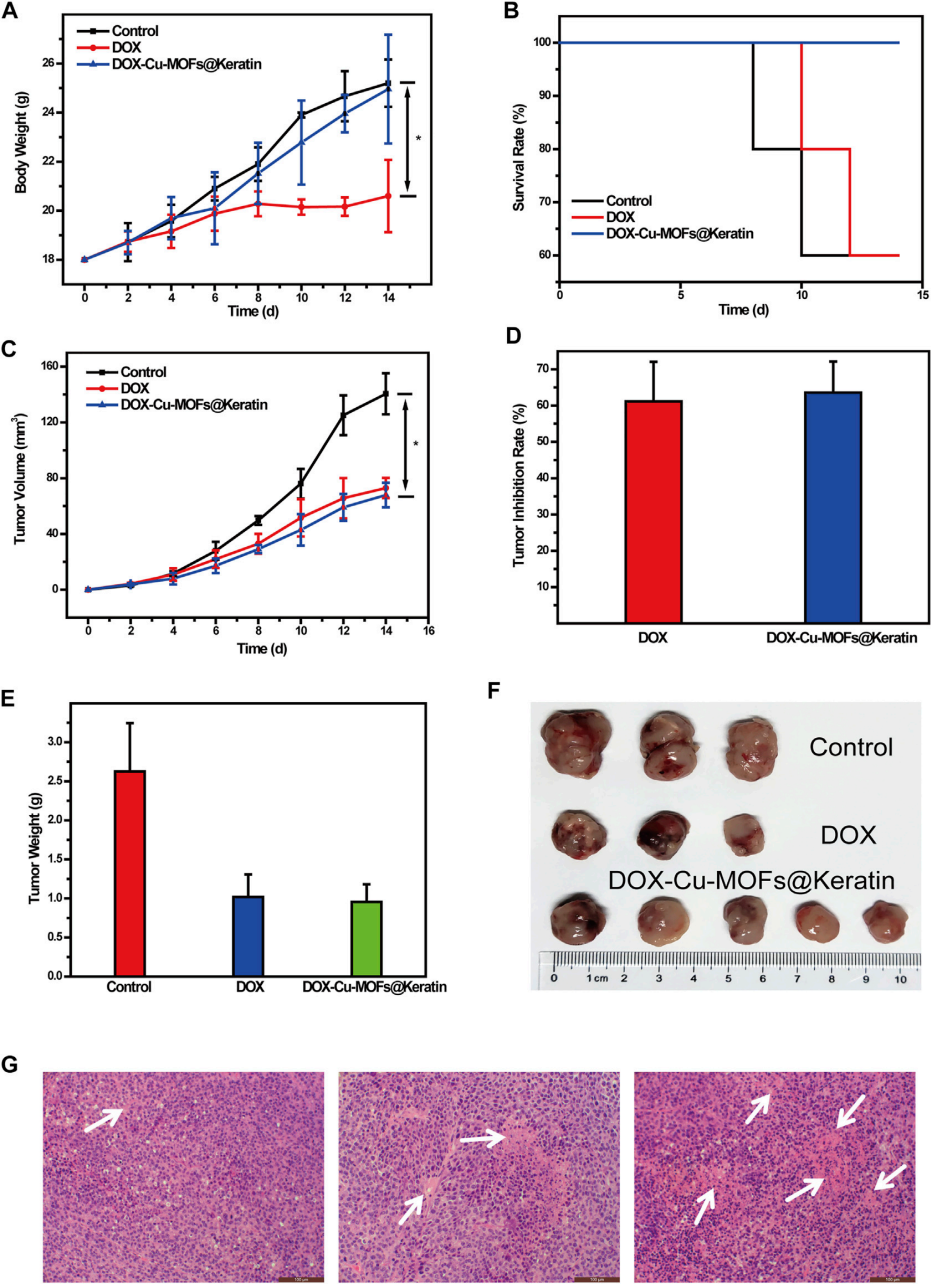
Anti-tumor efficacy of DOX-Cu-MOFs@Keratin on the tumor-bearing mice. **(A)** body weight of tumor-bearing mice (*n* = 5, mean ± SD); **(B)** survival rates of mice after the treatment; **(C)** tumor volume of the tumor-bearing mice; **(D)** tumor inhibition rate; **(E)** weight of the extracted tumors collected from different groups at the end day of the treatment; **(F)** photographs of tumors collected from different groups of mice at the end day of the treatment; and **(G)** H&E stained tumor tissues harvested from mice with different treatments.

To investigate the anti-tumor effects of DOX-Cu-MOFs@Keratin, free DOX and DOX-Cu-MOFs@Keratin were injected in mice bearing Hepa1-6 tumors. Systemic toxicity was evaluated by monitoring the body weight of the mice during the treatment regimen. As shown in Figure 8A, the mice treated with free DOX lost 20% of their body weight compared to that of the control group, while DOX-Cu-MOFs@Keratin injection resulted in negligible change in the body weight. In addition, while 40% of the tumor-bearing mice treated with saline or free DOX died at day 14, all animals in the DOX-Cu-MOFs@Keratin group were survived for 14 days (Figure 8B), confirming the low systemic toxicity of drug-loaded nanoparticles.

As shown in Figure 8C, the tumors grow rapidly in the mice treated with saline, but are suppressed by the DOX formulations. Compared to the control group, tumors of the free DOX and DOX-Cu-MOFs@Keratin groups were suppressed by 61.15% and 63.58%, respectively (Figure 8D). Accordingly, both the free DOX and DOX-Cu-MOFs@Keratin groups had smaller tumors with severe necrosis, as shown in Figures 8E,F,G. No significant differences were detected between the free DOX and DOX-Cu-MOFs@Keratin groups, possibly due to low H_2_O_2_ concentration in the tumor cells, which limited OH production and thus the effect of CDT (Li et al., 2022; Peng et al., 2022). Taken together, DOX encapsulation by Cu-MOFs@Keratin can reduce its systemic toxicity while retaining its anti-tumor efficacy.

## 4 Conclusion

We designed the multifunctional Cu-MOFs@Keratin nanoplatform for synergistic chemotherapy and chemodynamic therapy against tumor cells. The keratin coating improved the biocompatibility of the DOX-Cu-MOFs@Keratin, and released the loaded DOX in a sustained manner to adapt the low pH and high levels of GSH and trypsin in the TME. In addition, the DDS can also produce ROS (·OH) through cascade redox reactions, which achieved an ingenious GSH-triggered chemodynamic therapy. DOX-Cu-MOFs@Keratin can enter tumor cells through endocytosis, and exhibits therapeutic effects similar to that of free DOX in mice but causes lower systemic toxicity. As H_2_O_2_ concentration is low in the tumor cells, CDT has not achieved a satisfactory effect *in vivo*, which shall be the focus of our following work. Taken together, Cu-MOFs@Keratin could serve as a promising approach in cancer treatment.

## Data Availability

The original contributions presented in the study are included in the article/Supplementary Material, further inquiries can be directed to the corresponding authors.
