# Supplementary material for: Multi-stimuli responsive Cu-MOFs@Keratin drug delivery system for chemodynamic therapy
Source: Front Bioeng Biotechnol. 2023 Feb 2;11:1125348. doi: 10.3389/fbioe.2023.1125348 (PMC9936514; doi:10.3389/fbioe.2023.1125348)
Supplement: Supplementary file 1 [file DataSheet1.docx]

Supplementary Material

Multi-stimuli responsive Cu-MOFs@Keratin drug delivery system for chemodynamic therapy

Jinsong Du^1,2†^, Guanping Chen^3†^, Li Li^4^, Xinyi Yuan^1^, Jiang Yuan^2*^, Xiaoling Xu^1*^

*** Correspondence:** Jiang Yuan: jyuan@njnu.edu.cn; Xiaoling Xu: xuxl@hznu.edu.cn

# Characterization

Malvern Zeta sizer Nano ZS90 system (Malvern Co. Ltd., British) was applied to determine the size distribution range and the change of zeta potential of Cu-MOFs@Keratin in different environments using dynamic light scattering (DLS). Besides, the morphology characterization of Cu-MOFs and Cu-MOFs@Keratin was shot and observed by a transmission electron microscope (TEM H7650, Japan). The cytotoxicity of DOX-Cu-MOFs@Keratin against L929 cells and A549 cells was carried out by the MTT method. The cell uptake and intracellular drug release behavior of DOX-Cu-MOFs@Keratin were evaluated by confocal laser scanning microscopy (CLSM, Leica SP8).

# Stability in FBS

Cu-MOFs@Keratin was dissolved in RPMI-1640 medium, which contained 10% FBS, and was incubated in a constant temperature shock chamber at 37 ± 0.5 ℃ for 10 days. The changes of DDS’s particle size with Malvern Zetasizer Nano ZS90 were detected at different times.


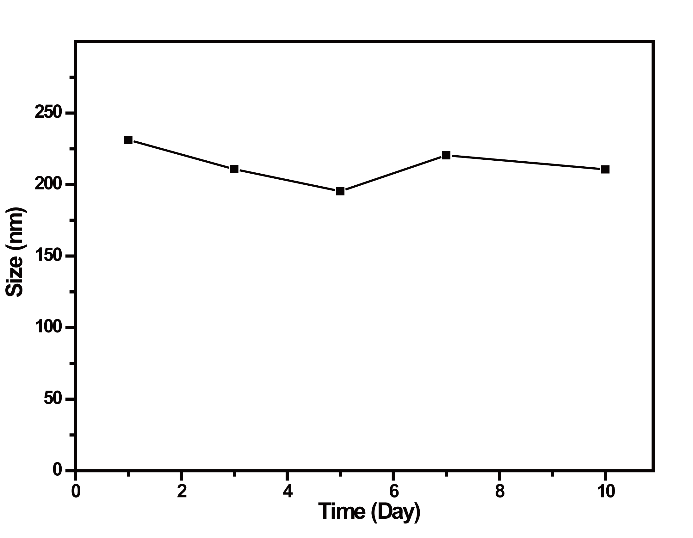


**Supplementary Figure 1.** Size changes of Cu-MOFs@Keratin in RPMI containing 10% FBS.

# Drug loading

**Figure S2** showed UV-visible spectrum of DOX, Cu-MOFs, DOX-Cu-MOFs and DOX-Cu-MOFs dissolved in 10 mM GSH. Compared with Cu-MOFs, Free DOX, DOX-Cu-MOFs and DOX-Cu-MOFs dissolved in 10 mM GSH all showed absorption peaks. The absorption peaks of DOX-Cu-MOFs that was dissolved in DI and that was dissolved in 10 mM GSH solution were 580 nm and 483 nm respectively. This may because loading DOX on Cu-MOFs triggered the offset of absorption peaks. The GSH solution of 10 mM can release DOX by destroying the Cu-MOFs structure in DOX-Cu-MOFs, which was consistent with the absorption peak of Free DOX. Above results indicated that the DOX was loaded to Cu-MOFs.


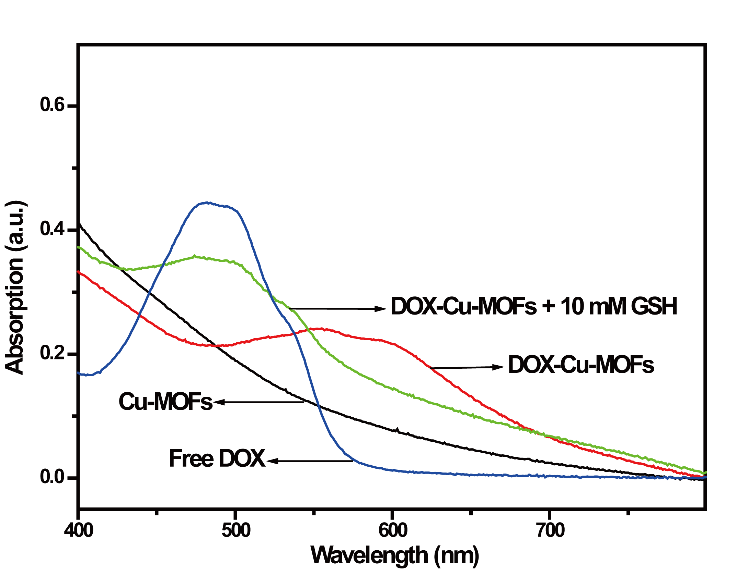


**Supplementary Figure 2.** UV-Vis spectra of Free DOX, Cu-MOFs, DOX-Cu-MOFs and DOX-Cu-MOFs in 10 mM GSH solution.

# Drug loading rate and encapsulation rate

The absorbance of supernatant obtained after centrifugation of drug-carrying nanoparticles DOX-Cu-MOFs@Keratin was measured at 483 nm by UV-VIS spectrophotometer. The drug loading rate and encapsulation rate were calculated according to **Figure S3** and its following formula.


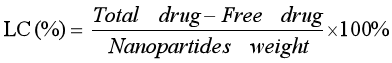

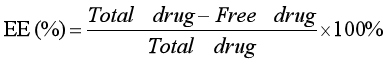


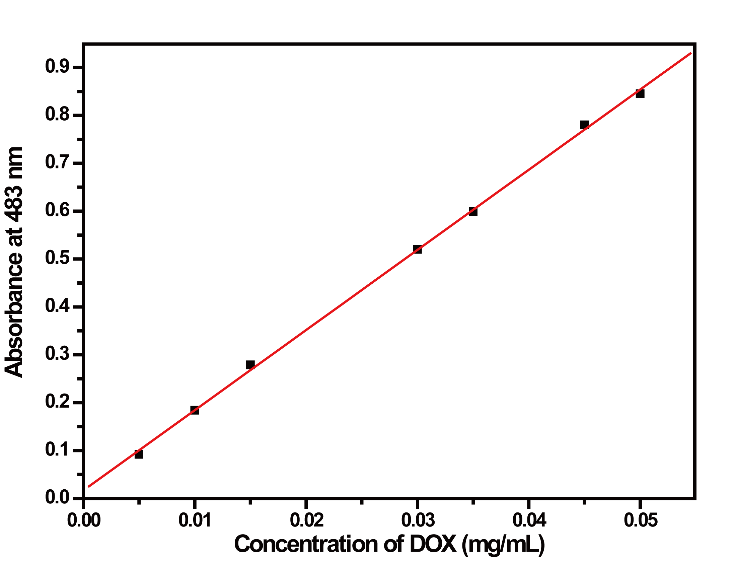


**Supplementary Figure 3.** Standard curve of DOX concentration and absorbanc.
